# The Modulation of PCSK9 and LDLR by Supercritical CO_2_ Extracts of *Mentha longifolia* and Isolated Piperitone Oxide, an *In Vitro* Study

**DOI:** 10.3390/molecules26133886

**Published:** 2021-06-25

**Authors:** Stefania Sut, Irene Ferrarese, Maria Giovanna Lupo, Nicola De Zordi, Elisa Tripicchio, Nicola Ferri, Stefano Dall’ Acqua

**Affiliations:** 1 Department of Pharmaceutical and Pharmacological Sciences, University of Padova, Via Marzolo 5, 35131 Padova, Italy; stefania.sut@unipd.it (S.S.); irene.ferrarese@unipd.it (I.F.); mariagiovanna.lupo@unipd.it (M.G.L.); nicola.ferri@unipd.it (N.F.); 2 Società Agricola Moldoi – S.A.M, SrL, Loc. Maras Moldoi 151/a, 32037 Sospirolo, Italy; ndezordi@gmail.com (N.D.Z.); aziendamoldoi@gmail.com (E.T.)

**Keywords:** *Menta longifolia*, nutraceutical, phytosterols, fatty acids, piperitone oxide, terpenoids, PCSK9, LDLR

## Abstract

In the present study the ability of supercritical carbon dioxide (SCO_2_) extracts of *M. longifolia* L. leaves to modulate low-density lipoprotein receptor (LDLR) and proprotein convertase subtilisin/kexin type 9 (PCSK9) expression was evaluated in cultured human hepatoma cell lines Huh7 and HepG2. Two SCO_2_ extracts, one oil (ML-SCO_2_) and a semisolid (MW-SCO_2_), were subjected to detailed chemical characterization by mono- and bidimensional nuclear magnetic resonance (1D, 2D-NMR), gas chromatography coupled with mass spectrometry (GC-MS) and liquid chromatography coupled with mass spectrometry (LC-MS). Chemical analysis revealed significant amounts of fatty acids, phytosterols and terpenoids. ML-SCO_2_ was able to induce LDLR expression at a dose of 60 µg/mL in HuH7 and HepG2 cell lines. Furthermore, ML-SCO_2_ reduced PCSK9 secretion in a concentration-dependent manner in both cell lines. Piperitone oxide, the most abundant compound of the volatile constituent of ML-SCO_2_ (27% *w/w*), was isolated and tested for the same targets, showing a very effective reduction of PCSK9 expression. The overall results revealed the opportunity to obtain a new nutraceutical ingredient with a high amount of phytosterols and terpenoids using the SCO_2_ extraction of *M. longifolia* L., a very well-known botanical species used as food. Furthermore, for the first time we report the high activity of piperitone oxide in the reduction of PCSK9 expression.

## 1. Introduction

*Mentha* species are commonly used in food and as traditional herbal medicine all over the world, and many different varieties are considered of high value both for culinary and therapeutic uses. *Mentha longifolia* L. (Lamiaceae (Labiatae)), referred to as wild mint or horse mint, is widely diffused in Europe, Asia and non-tropical areas of Africa [1,2,3]. In Italy, it can be found in many regions, mostly in the alpine part in the area from 900 to 2000 mt. Related to the ethnomedicinal uses, the species is mostly used for treatment of gastrointestinal, respiratory and inflammatory diseases, as well as menstrual pain [1]. All these uses are common in different cultures all over the world, indicating the potential medicinal usefulness of its constituents. Phytochemical composition of the plant is well-studied and shows the presence of ceramides, cinnamate, flavonols, monoterpene and sesquiterpene [1,2]. Nutraceuticals have been proposed as key tools for the prevention of some degenerative diseases and especially for health promotion [4,5,6], and there is a large interest among consumers, especially thanks to the claimed versatility of natural compounds in maintaining health status. Nowadays, red yeast rice, berberine, plant sterols, dietary fibers, grape and other vegetable are considered the most important studied supplements with properties of lipid control [5]. Indeed, lifelong exposure to high blood cholesterol levels has been found to be the leading culprit for atherosclerotic CVD (ASCVD) onset and progression [7], nowadays recognized as the leading cause of death worldwide. The possibility of implementing a non-pharmacologically based treatment for hypercholesterolemia is thus getting increasing attention, and it is considered to be an important preventive action to take when hypercholesterolemia is mild or moderate. Such an opportunity can be valuable when low and/or moderate risk factors for ASCVD development are present, or when the patient shows drug intolerance [8]. Furthermore, the possibility of using nutraceuticals in this area can be valuable, especially in cases when the patient fails to reach the total cholesterol (TC) and low-density lipoprotein (LDL) cholesterol (LDL-C) targets despite the maximal tolerated dose of the most widespread hypocholesterolemic drugs, such as statins and ezetimibe [9]. The significant role of the cholesterol-lowering nutraceuticals can be referred to the presence of statin-like products based on red yeast rice; nevertheless, the possibility of significant side effects raises questions on the doses to be used in food supplements and nutraceuticals. The European Food Safety Authority (EFSA) opinion on the safety of red yeast rice indicates an uncertainty about the definition of a dietary intake that does not give rise to concerns about potential harmful effects [10].

The statin-based treatments are largely diffused, and risks and side effects are present and can be relevant, reducing compliance and treatment efficacy. Based on these premises, there is an urgent need to develop more effective hypolipidemic agents that originate from natural products, and the nutraceuticals in this field are growing at a high-speed, influencing marketing and consumer choices.

Essential oils from various sources have been studied for their effects in cardiovascular diseases, including improvements in lipid balance, liver and endothelial functions and reductions in blood pressure, oxidative stress, thrombosis and inflammation. Some reports indicate that some essential oils promote vascular relaxation and inhibit diabetes development and angiogenesis. Therefore, essential oils and their active components may be promising therapeutic agents for CVDs [11]. Thus, the search for new lipid-lowering agents can start from essential oils. As an example lemon and lime were studied *in vivo* models for their cholesterol-lowering properties [12,13]. Also, eugenol, one of the most diffused compounds in several essential oils, was studied for its lipid-lowering effects and results indicated that eugenol does not inhibit β-hydroxy β-methylglutaryl-CoA (HMG-CoA) reductase but rather induces its action by interaction with TRPV1 channels [13].

Essential oils are complex mixtures of volatile constituents, in many cases composed of monoterpenes and sesquiterpene, which can be found in diffused vegetable and spice [11]. Such mixtures are usually extracted by hydro-distillation. In the development of new ingredients, supercritical CO_2_ (SCO_2_) extraction can be a valuable alternative technique for the extraction of such compounds because it presents advantages compared to hydro-distillation due to lower temperature operation, and it is safer compared to other extraction approaches because it avoids the use of solvents. SCO_2_ extraction can be considered a strategy to extract natural compounds with environmental friendliness. In fact, it is a green extractive approach that avoids the use of solvents that lead to extracts that present different compositions compared to traditional hydroalcoholic or aqueous extraction, such as maceration or infusion/decoction. Furthermore, the technique has allowed the extraction of lipophilic fractions in mild temperature conditions [14].

To our knowledge, no information is available related to the possible effects of *M. longifolia* L. extracts as active ingredients in nutraceuticals for their cholesterol-lowering effects. This plant can be a good candidate in the search of compounds with cholesterol-lowering activities due to its complex phytochemical composition and common use in traditional medicine and as a food, suggesting its safety.

The aim of this study was the evaluation of the SCO_2_ extract of *M. longifolia* L. as a novel nutraceutical ingredient with potential effects on cholesterol. Furthermore, the detailed phytochemical compositions of two different extracts were obtained by combining various analytical techniques. Extracts were tested in two key targets players, LDL receptor (LDLR) and proprotein convertase subtilisin/kexin type 9 (PCSK9), in two human hepatoma cell lines (Huh7 and HepG2), to investigate the *in vitro* ability to modulate cholesterol metabolism [15,16]. To assess the potential role of the main constituent, piperitone oxide was isolated and tested in the same cellular model.

## 2. Results

From the aerial parts of *M. longifolia* L., two different fractions were obtained using SCO_2_ with a two-stage apparatus. The first fraction appeared as semisolid wax (MW-SCO_2_), the second as an oil (ML-SCO_2_). The extracts were analyzed using nuclear magnetic resonance (NMR), liquid chromatography atmospheric pressure chemical ionization mass spectrometry (LC-APCI-MS) and gas chromatography mass spectrometry (GC-MS) approaches. NMR allowed the rapid identification of the main metabolites and the detection of non-volatile constituents. LC-APCI-MS was used to obtain the chemical profile and quantitative data about phytosterols. GC-MS was used to analyze fatty acid composition after derivatization to methyl esters and to study volatile constituents.

### 2.1. NMR Analysis of Extracts

**Figure 1:**
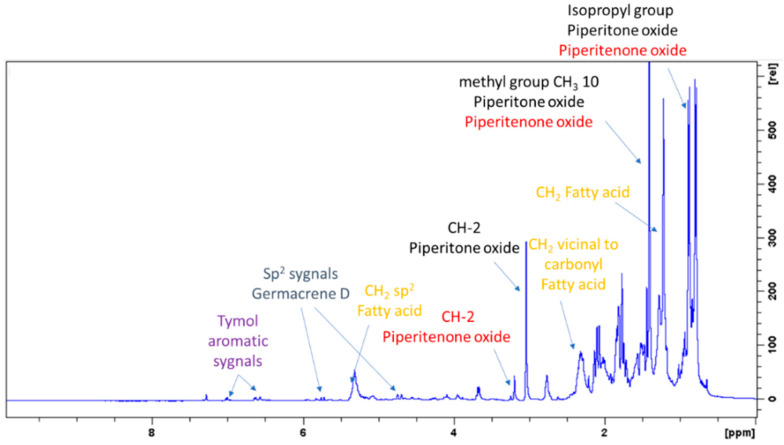
^1^H NMR (400 MHz) of ML-SCO_2_. Principal assignments are highlighted: piperitone oxide (black), piperitenone oxide (red), germacrene D (light blue), tymol (purple), fatty acid (yellow).

**Figure 2:**
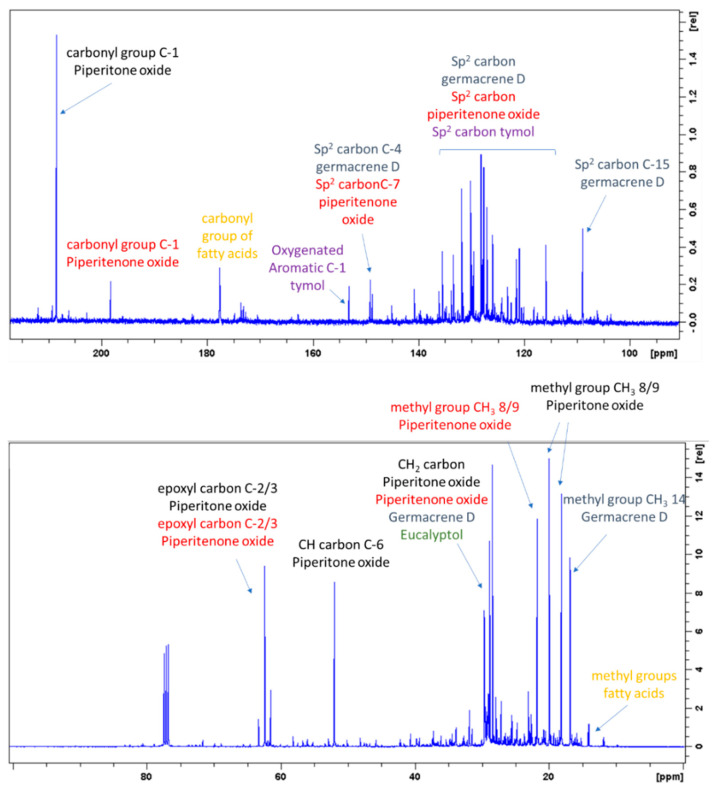
^13^C NMR of ML-SCO_2_ in the range 220–90 ppm (above) and in the range of 100–0 ppm (below). Principal assignments are highlighted: piperitone oxide (black), piperitenone oxide (red), germacrene D (light blue), tymol (purple), eucalyptol (green) and fatty acid (yellow).

The ^1^H-NMR spectra of the two extracts (ML-SCO_2_ and MLW-SCO_2_) were similar and presented signals due to fatty acids and other aliphatic species. Assignments of the different compounds were obtained on the basis of the combination of the data obtained from ^13^C-NMR, HSQC-DEPT, HMBC and COSY experiments performed on the ML-SCO_2_ sample and comparing them with those from the literature and databases. Exemplificative ^1^H-NMR and ^13^C-NMR spectra of ML-SCO_2_ are shown in Figure 1 and Figure 2. The two samples presented several signals in the aliphatic region that could be ascribed to different monoterpenoid and sesquiterpene derivatives. A supercritical CO_2_ extraction approach made it possible to obtain not only the mono- and sesquiterpene volatile fractions but also non-volatile constituents. The main assignments are summarized in Table 1, showing the structure of the five most abundant constituents that were identified based on their NMR data.

^1^H-NMR showed crowded signals in the aliphatic region (0–2 ppm) and further signals were detected in the spectral region at δ 2.5–6.0 (Figure 2). The assignments of the isopropyl moiety of piperitone oxide and of the isopropylen moiety of piperitenone oxide were confirmed by 2D data, allowing the identification of the two compounds in the extract. Signals ascribable to geminal methyl groups of piperitone oxide were detected at δ 0.90 (δ_C_ 20) and δ 0.80 (δ_C_ 18) and showed HMBC correlation with carbon resonance at δ 52 (C-6) and 29 (C-7), while the methyl C-8 showed HMBC correlation with carbon resonance at δ 18 and the methyl C-9 with carbon resonance at δ 20, respectively. Concerning piperitenone oxide, signals of methyl groups at δ 2.07 (δ_C_ 23.3) and δ 1.77 (δ_C_ 23.3) showed HMBC correlations with carbon resonance at δ 149 (C-7), 129 (C-6) and 23.3(C-8/9), confirming the presence of sp^2^ carbons of the isopropylene moiety. The signal at δ 3.03 (δ_C_ 62) assigned to C-2 of piperitone oxide showed HMBC correlations with C-1 at δ 208.5, C-3 at δ 61.5, C-6 at δ 52 and C-10 at δ 21.5, confirming the structure. Signal H-2 of the epoxide moiety of piperitenone oxide at δ 3.20 (δ_C_ 63.2) showed HMBC correlations with carbon resonance at δ 198 (C-1), 129 (C-6), 63.1 (C-3) and 21.7 (C-10), confirming the structure of the compound.

Proton signals at δ 4.65, 4.75, 5.21 and 5.77 indicated the presence of sp^2^ protons of germacrene D (Figure 2), namely CH_2_-15, CH-6 and CH-5, as indicated in the Table 1. The sp^2^ CH_2_-15 of germacrene D was detected in HSCQ-DEPT at δ 4.65–4.75 (δ_C_ 109) and diagnostic HMBC correlations were observed from this signal with carbon resonances at δ 149 (C-4), 135 (C-5) and 35 (C-3). HMBC correlations were observed from CH-6 at δ 5.77 (δ_C_ 135) with carbon resonances at δ 149, δ 52 and δ 35; other long-range HMBC correlations were observed from CH-5 at δ 5.21 (δ_C_ 133), with carbon resonance at δ149 (C-4), 52 (C-7), 109 (C15) and 35 (C-3), confirming the presence of a conjugated double bond with the methylene moiety and thus supporting the identification of germacrene D.

In the aromatic region signals at δ 7.01, 6.64 and 6.59 (brs) were assigned to thymol (Figure 2). Signals at δ 6.59 (δ_C_ 115) (brs), δ 6.64 (δ_C_ 121.1) (d, J = 7.55) and δ 7.01 (δ_C_ 126) (d, J = 7.55) were all part of a single spin system, as shown in COSY. HMBC correlations are summarized in Table 2, confirming the presence of trisubstituted benzene rings in positions 1, 3 and 6. Deshielded carbon at δ 153.5 revealed a hydroxyl aromatic moiety in position 2. Signals of the benzyl methyl group at δ1.19 (δ_C_ 22.4) showed HMBC correlations with carbon resonances at δ 131.5 and δ 26.0, confirming the presence of the isopropyl moiety. Eucalyptol in the mixture was detected thanks to the diagnostic signals, where the signal at δ 1.03 (δ_C_ 27.8) was assigned to the methyl group that presented HMBC correlations with resonances at δ 69.8 and 31.3.

NMR investigation made it possible to obtain a fingerprint of the extract confirming the presence of fatty acid, monoterpene and sesquiterpenoid as main constituents.

### 2.2. GC-MS of Volatile Constituents

To complete the characterization of the extracts, GC-MS analysis was performed due to its sensitivity and specificity. The GC-MS analysis made it possible to obtain quali–quantitative data, Compounds were identified based on the calculation of the Kovats index as well as by comparison of EI mass spectra with databases and results from the literature; all data are summarized in Table 2. The analysis allowed the identification of 26 compounds that were quantified using an internal standard. Comparing the total amount of volatile constituents in ML-SCO2 and MLW-SCO_2_, we observed values of 39 and 24%, respectively, indicating a significant presence of non-volatile compounds.

The most abundant volatile compounds in both the extracts were piperitone oxide, piperitenone oxide and germacrene D at 26.59%, 3.77% and 3.08% in ML-SCO_2_ and 16.49%, 1.86% and 2.6% in MLW-SCO_2_, respectively, as reported in Table 2.

### 2.3. Quantification of Fatty Acid Methyl Esters

The NMR data indicated the presence of lipids in both the extracts. To establish lipid composition, GC-MS of fatty acid methyl esters after derivatization was used, and palmitic acid (2.84%) and linoleic acid (2.18%) resulted in the most abundant fatty acids in ML-SCO_2_, while alpha-linolenic acid (9.39%) and palmitic acid (3.68%) were the most abundant in MLW-SCO_2_, which presented almost the same percentage of linoleic acid (2.81%). The overall contents of fatty acid were 8% and 18% for ML-SCO2 and MLW-SCO_2_, respectively (Table 3).

### 2.4. Quantification of Phytosterols

In the different classes of lipophilic compounds derived from plant sources, phytosterols play an important role due to their claimed effects on health. Phytosterols can be revealed by NMR only if they are present in large amounts and their diagnostic signals can be overlapped to other constituents. Furthermore, their volatility is poor, and thus they cannot be analyzed by GC without derivatization. Due to their physic-chemical properties, their lipophilic nature makes them difficult to analyze using electro-spray ion sources. For this reason, an LC-APCI-MS approach was used to obtain quali–quantitative fingerprints, using a previously published approach that we have used for other analyses of plant lipids [17]. The results showed the presence of β-sitosterol and stigmasterol as the main constituents at concentrations of 5.57 and 0.99 mg/g in MLW-SCO_2_ and 0.31 and 0.05 in ML-SCO_2_, respectively. Other minor identified constituents, on the basis of their molecular weight and mass fragmentation, were gamma-sitosterol, brassicasterol and cycloartanol; the results are summarized in Table 4. With regard to the total phytosterol in the extracts, MLW-SCO_2_ contained a higher amount compared to ML-SCO2 at 7.50 vs. 0.41 mg/g.

### 2.5. Effect of Mint Extracts on LDLR and PCSK9 Expression in Huh7 Cell Line

**Figure 3:**
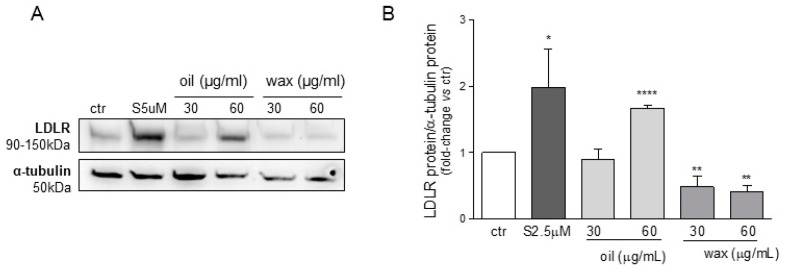
Effects of mint oil (ML-SCO_2_) and wax (MLW-SCO_2_) extracts on LDLR expression. Huh7 cells were treated for 72 h with mint oil and wax extracts at the indicated concentrations. Protein expression of the LDL receptor (**A**,**B**) was evaluated through an ECL-based Western blot assay by loading 20 μg of protein per sample. Alpha-tubulin was used as housekeeping normalizer. Simvastatin 5 μM served as a positive control. Data are presented as means ± SD of three independent experiments. * = *p* < 0.05, ** = *p* < 0.01, **** = *p* < 0.0001 vs. control (ctr).

In a first series of experiments, we tested the effects of the extracts named as oil (ML-SCO_2_) and wax (MLW-SCO_2_) on the expression of the LDLR in Huh7 cells. Cells were thus incubated with two concentrations of mint oil and wax (30 and 60 µg/mL) for 72 h in MEM/0.4%FBS and using simvastatin 5 µM as a positive control. Under these experimental conditions, we did not observe any significant cytotoxic effects for either oil or wax extracts (data not shown). As shown in Figure 3, only minimum oil induced a significant upregulation of the LDLR at 60 µg/mL, while wax slightly reduced its expression.

**Figure 4:**
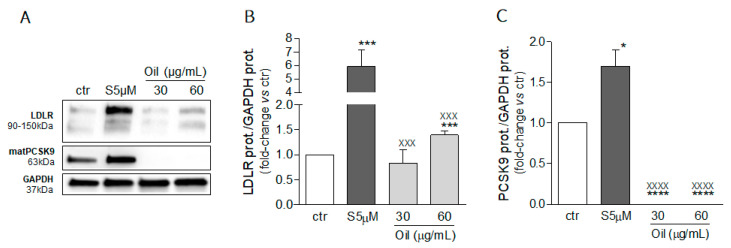
Effect of mint oil extract (ML-SCO_2_) on LDLR and PCSK9 protein expression. Huh7 cells were treated for 72 h with mint oil extract at the indicated concentrations. Protein expression of LDL receptor (**A**,**B**) and PCSK9 (**A**,**C**) was evaluated through an ECL-based Western blot assay by loading 20 μg of protein per sample. GAPDH was used as housekeeping normalizer. Simvastatin 5 μM served as a positive control. Data are presented as means ± SD of three independent experiments. * = *p* < 0.05, *** = *p* < 0.001, **** = *p* < 0.0001 vs. ctr; xxx = *p* < 0.001, xxxx = *p* < 0.0001 vs. Simvastatin 5 µM (S5 µM). matPCSK9: mature form of PCSK9; ctr: control

From this analysis, we focused our attention on the mint oil extract ML-SCO_2_ and determined its effect on both LDLR and PCSK9 protein levels by Western blotting analysis from total cell lysates. As shown in Figure 4A,B, mint oil produced a significant increase (50%) in LDLR expression compared to the basal condition (*p* < 0.001), when tested at 60 µg/mL. Conversely, the same experimental settings determined an almost complete suppression of PCSK9 levels both at 30 and 60 µg/mL of mint oil (Figure 4A,C; *p* < 0.0001 vs. ctr).

**Figure 5:**
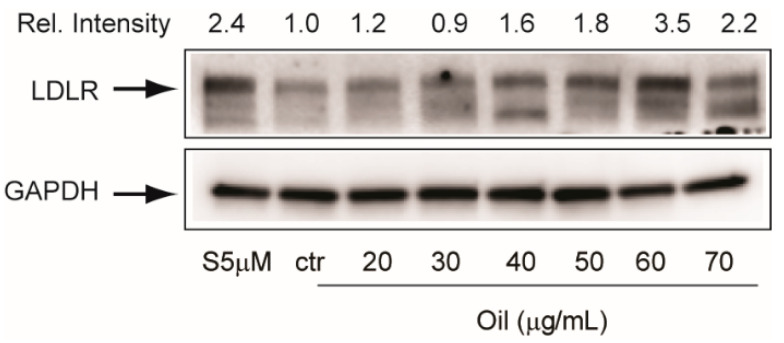
Effect of mint oil extract (ML-SCO_2_) on LDLR protein expression in HepG2 cells. Experimental conditions were the same as described for Figure 4. Rel. Intensity: relative intensity of the LDLR normalized with GAPDH.

The effect of mint oil was also confirmed in HepG2 cells with a concentration-dependent increase of LDLR expression, reaching a maximal effect at 60 µg/mL (Figure 5).

**Figure 6:**
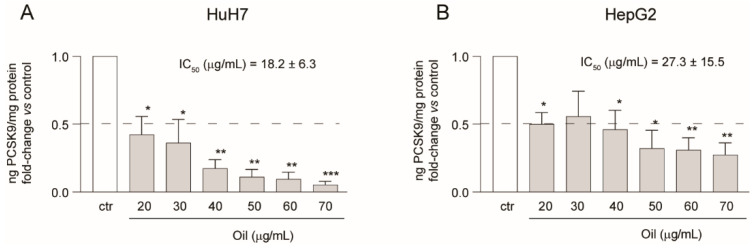
Effect of mint oil extract on PCSK9 secretion in Huh7 and HepG2 cell lines. Huh7 (**A**) and HepG2 (**B**) cells were treated for 72 h with mint oil at the indicated concentrations. DMSO samples were used as control (ctr). PCSK9 secretion in the culture media was then evaluated by ELISA assay and normalized with cell protein concentrations. The ratios were plotted as fold-change vs. control (set as 1); 50% PCSK9 inhibition is represented by the black dashed line. Data are presented as means ± SD of three independent experiments. * *p* < 0.05, ** *p* < 0.01, ***0.001 vs. control.

Having observed a very strong inhibition on PCSK9 expression, we decided to further explore the activity of mint oil extract on this relevant pharmacological target by measuring the extracellular concentrations in two different cell lines, Huh7 and HepG2. As shown in Figure 6 the effect of mint oil on the secreted form of PCSK9, determined by ELISA assay from conditioned media, was very similar in both cell lines. The concentration-dependent effect was determined between 20 µg/mL and 70 µg/mL. After 72 h of incubation, we observed a concentration-dependent inhibition of PCSK9 with IC_50_ values of 18.2 ± 6.3 µg/mL in the Huh7 cell line and 27.3 ± 15.5 µg/mL in the HepG2 cells (Figure 6).

### 2.6. Piperitone Oxide Decreases Both LDLR and PCSK9 Protein Levels in Huh7 Cell Line

**Figure 7:**
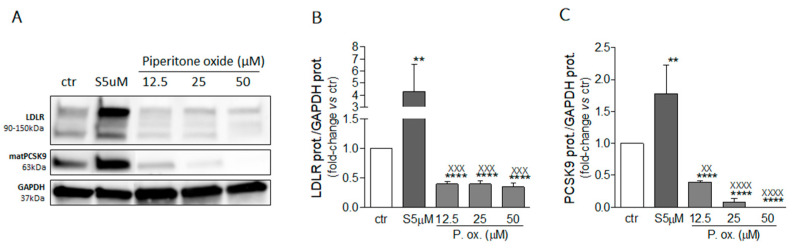
Effect of piperitone oxide on LDLR and PCSK9 protein expression. Huh7 cells were treated for 72 h with piperitone oxide (P. ox.) at the indicated concentrations. Protein expressions of the LDL receptor (**A**, **B**) and PCSK9 (**A**, **C**) were evaluated through an ECL-based Western blot assay by loading 20 μg of protein per sample. GAPDH was used as housekeeping normalizer. Simvastatin 5 μM was used as a positive control. Data are presented as means ± SD of three independent experiments. ** = *p* < 0.01, **** = *p* < 0.0001 vs. ctr; xx = *p* < 0.01, xxx = *p* < 0.001, xxxx = *p* < 0.0001 vs. Simva 5 µM (S5 µM). matPCSK9: mature form of PCSK9.

To identify one of the possible active components of mint oil, we investigated the effect of piperitone oxide, the main volatile component of mint oil (26.6%) extract (Figure 1; Table 2). Piperitone oxide already significantly reduced PCSK9 intracellular protein levels by 60% at the lowest tested concentration of 12.5 µM compared to control (*p* < 0.0001), with the strongest effect at 25 µM (−92%, *p* < 0.001 vs. control), whilst no PCSK9 signal was detected at 50 µM (*p* < 0.0001 vs. control) (Figure 7A,C). Conversely, the effect on LDLR was intensive but not concentration-dependent (−60% each, *p* < 0.0001 vs. control) (Figure 7A,B).

### 2.7. Simvastatin Can Rescue the Piperitone Oxide Effect on LDLR and PCSK9 Expression in Huh7 Cell Line

**Figure 8:**
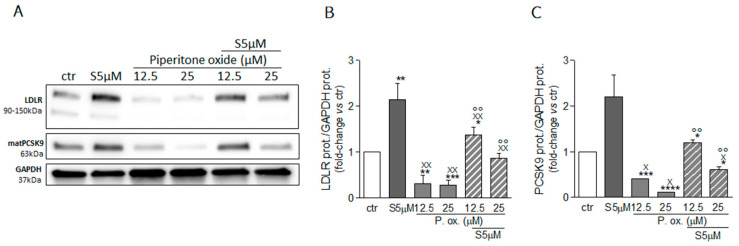
Effect of simvastatin and piperitone oxide combination on LDLR and PCSK9 protein expression. Huh7 cells were treated for 72 h with simvastatin (S5 µM) or piperitone oxide (P. ox.), alone or in combination with each other, at the indicated concentrations. Protein expression of LDL receptor (**A**,**B**) and PCSK9 (**A**,**C**) was evaluated through an ECL-based Western blot assay by loading 20 μg of protein per sample. GAPDH was used as housekeeping normalizer. Simvastatin 5 μM was used as a positive control. Data are presented as means ± SD of three independent experiments. * = *p* < 0.05, ** = *p* < 0.01, *** = *p* < 0.001, **** = *p* < 0.0001 vs. ctr; xxx = *p* < 0.001, xxxx = *p* < 0.0001 vs. Simva 5 µM (S5 µM); °°°° = *p* < 0.0001 vs. P. ox. 25 µM. matPCSK9: mature form of PCSK9.

The present data indicate a possible negative effect of piperitone oxide on the sterol regulatory element-binding protein 2 (SREBP2) pathway responsible for LDLR and PCSK9 gene transcription. For this reason, we performed a new series of experiments with simvastatin, the HMG-CoA reductase inhibitor and inducer of SREBP activation. As predicted, simvastatin induced a significant increase both in LDLR (+113%) and PCSK9 (+129%) expression compared to vehicle-treated cells (Figure 8A), while piperitone oxide confirmed its significant SREBP2 pathway suppressing activity, reducing the expression of both proteins (Figure 8). The addition of simvastatin to piperitone oxide was sufficient to rescue the effect of this latter, with results comparable to those obtained in control cells when combined with piperitone oxide 12.5 µM (Figure 8).

The two extracts were assayed for their ability to influence LDLR in the Huh7 cell line to further assess the potential mechanism of action related to lipid control activity. Only ML-SCO2 was significantly active and was able to induce LDLR expression.

To further explore a possible effect of ML-SCO_2_ on lipid metabolism, we carried out a series of experiments with the aim of determining the effect and the molecular mechanism underlying the effect on the LDLR. We focused our attention on PCSK9, a soluble protein capable of inducing the degradation of the LDLR and thus negatively controlling the cholesterol uptake by the liver [16]. For the positive effect of ML-SCO_2_ on the expression of the LDLR, we observed a concomitant complete suppression of PCSK9 levels, thus indicating a berberine-like behavior [18]. This effect may contribute to the induction of the LDLR by the same extract.

We then tested the effect of the minimum oil main component, piperitone oxide, and observed a very effective and concentration-dependent decrease of both PCSK9 and LDLR expression, suggesting an inhibitory action on the SREBP2 pathway. Indeed, the addition of simvastatin, an enhancer of the SREBP2 cascade, to piperitone oxide rescued the effect on LDLR and PCSK9. Further experiments are needed to better clarify the involvement of the SREBP2 pathway due to piperitone oxide treatment. However, it should be noted that PCSK9 expression is more sensitive to piperitone oxide treatment compared to LDLR, thus opening up to a possible effect on additional transcription factors involved in its transcription, including HNF-1α [19].

## 3. Discussion

*M. longifolia* L. possesses a rich phytocomplex characterized by monoterpene and sesquiterpenoids, and SCO_2_ extraction represents an innovative strategy to obtain extracts enriched with bioactive compounds. In this study, two different extracts were obtained using SCO_2_ from the aerial parts of *M. longifolia*. The two extracts presented different physical appearance, the first being an oil and the second a semisolid residue. The composition of the extracts was studied using different approaches, namely NMR, GC-MS and LC-MS, allowing complete phytochemical characterization. The NMR data agree with the GC-MS results (Table 2), indicating piperitone oxide, piperitenone oxide, thymol, eucalyptol and germacrene D as the most abundant terpenoid constituents in the extracts.

SCO_2_ extraction is a technology that allows extraction of a wide range of diverse compounds from a variety of plant matrices. It is suitable for extraction with many non-polar to moderately polar compounds. Supercritical fluid extraction is considered helpful due to its improved speed and selectivity and the mild temperature condition preserves thermolabile compounds and product characteristics [14]. The two obtained fractions, ML-SCO_2_ and MLW-SCO_2_, were formed of lipophilic and volatile constituents of the plant. To our knowledge, limited information is available related to SCO_2_ extraction of *M. longifolia*. Thus, for comparison purposes, we considered the literature related to the essential oil composition of *M. longifolia*. A previous paper reported the chemical characterization of essential oil obtained by distillation and the main volatile constituents were menthone (19.31%), pulegone (12.42%), piperitone (11.05%) and dihydrocarvone (8.32%) [3]. Other work reported results similar to our data, finding that the major compounds in essential oil were *cis*-piperitone epoxide (from 7.8% to 77.6%), and piperitenone oxide (from 1.5% to 49.1%) [20]. A previous paper described supercritical extraction from *Mentha spicata* species and concluded that SCO_2_ can be selectively used to efficiently extract oil, with extracts containing higher amounts of carvone compared to hydro-distillation [21]. More recently, a comparison of SCO_2_ extraction, steam distillation and solvent extraction was performed on *M. longifolia* collected in China. Volatile constituents were assessed and the main compounds were found to be limonene and carvone [22]**,** thus demonstrating a strong difference in composition from our sample.

With regard to the fatty acid composition, palmitic (2.84%) and linoleic (2.18%) acids were the most abundant fatty acids in ML-SCO_2_, while alpha-linolenic (9.39%), palmitic (3.68%) and linoleic (2.81%) acids were the most abundant in MLW-SCO_2_. The amount of β-sitosterol was 0.31 mg/g and 5.6 mg/g in ML-SCO_2_ and MLW-SCO_2_, respectively. SCO_2_ extraction can be used to obtain an enriched phytosterol fraction, as was demonstrated by the fact that the phytosterol concentration in MLW-SCO_2_ was 18 times higher than that inn ML-SCO_2_. Campesterol, stigmasterol and β-Sitosterol were previously reported in *M. longifolia* in petroleum ether extract [2]. The wax fraction MLW-SCO_2_ presented 0.75% total phytosterols while the oil fraction ML-SCO_2_ presented a much lower concentration.

The identification of phytosterol in *M. longifolia* extracts in the present study can be considered valuable due to the description of the activity of these compounds as cholesterol-lowering agents and for cardiovascular disease prevention. Thus, the supercritical extracts obtained from *M. longifolia* could be a good starting material to produce novel nutraceutical formulations due to their unsaturated fatty acid, phytosterol and terpenoid contents. Such ingredients may be useful in lipid metabolism due to the mechanism of action claimed for phytosterols; that is, their possible competition with intestinal cholesterol for incorporation into micelles. Phytosterols may act as effective triglyceride-lowering agents in hypertriglyceridemic subjects and have cholesterol-lowering capabilities [23]. The absolute effectiveness of phytosterol-mediated cholesterol lowering is affected by a variety of factors including dose, intake frequency and individual baseline cholesterol concentrations. Clinical trials have usually been performed with the administration of 0.6 to 3 g per day of phytosterols. With regard to the possible use of *Mentha* extracts as nutraceuticals, the presence of this active compound in lipid control could contribute to helping and explaining different mechanisms of action. One study reported an investigation into the interaction of phytosterols with the hepatic LDL receptor in *apolipoprotein E*-knockout mice in comparison with wild-type mice, and it was found that treatment did not alter receptor function. Phytosterols significantly increased fecal sterol excretion and decreased hepatic cholesterol concentrations [24]. Due to the higher phytosterol content, MW-SCO_2_ can be considered a valuable nutraceutical source of phytosterols.

Overall, the present study suggests the potential usefulness of *M. longifolia* supercritical extracts, paving the way for in-depth analyses aimed at developing their use as a new natural extract with hypocholesterolemic properties, an effect that needs to be confirmed *in vivo*.

## 4. Materials and Methods

### 4.1. Plant Material

The plant material was collected in the fields of S.A.M. srl, located in Sospirolo Belluno (Italy), in summer 2019. Taxonomic identification was performed by one of the authors (N.De Zordi) and a voucher specimen was deposited at the Natural Product Lab of the Department of Pharmaceutical and Pharmacological Sciences, Padova University (ML2020A). The aerial part materials were dried in a cool desiccator (NWT-35, Italy). After drying at 35 °C until the mint reached 5.5% of the residual humidity, the plant materials were stored in shade at 20 °C.

### 4.2. Supercritical CO_2_ (SCO_2_) Extraction

**Figure 9:**
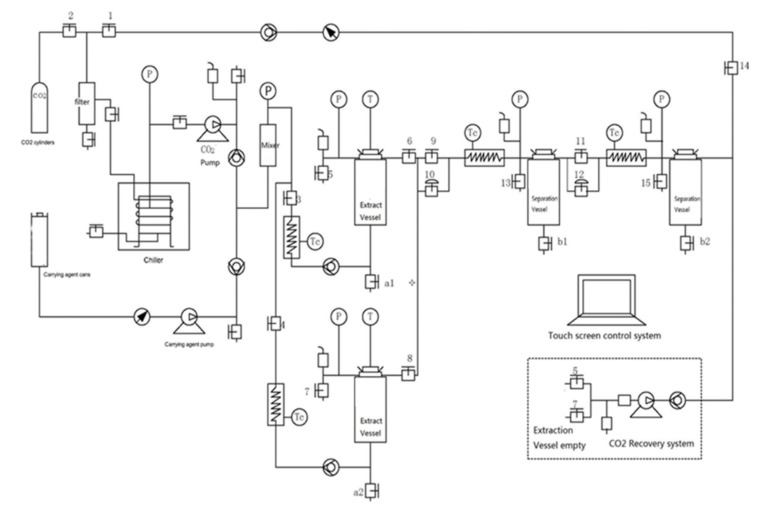
Supercritical extraction equipment. P = pressure controller, T = temperature controller, Tc = heater exchanger.

Supercritical extraction of *M. longifolia* L. was performed with a TH22-10 x2 supercritical CO_2_ extraction apparatus (Toption Instrument Co. Ltd, YanTa District, Xi’an, China), depicted in Figure 9. Briefly, the plant was equipped with two extraction vessels of 10 L and two separators of 5 L. The carbon dioxide (Siad SpA, Trieste, Italy; 99.99% purity, food grade) was carried with a high-pressure liquid pump (Toption Instrument Co. Ltd YanTa District, Xi’an, China).

First, 2.9 kg of milled *M. longifolia* (≤40 mesh) was weighed into the stainless-steel extraction basket, which was loaded onto the jacketed extraction vessel. The flow rate of supercritical solvent was set at 1 L/min in all experiments. The extraction pressure was set to 150 bar, while the extraction temperature was set at 40 °C. The first separator was operated at 70 bar and 45 °C and the second one at 45 bar and 40 °C. The extraction was carried on until the amount of extract collected over 1 h decreased to under 0.1% of the raw material. During the supercritical carbon dioxide extraction, water (bound moisture from plant material) was co-extracted, then decanted, and the crude extract was collected and stored. The crude extracts were weighed, and the yield was calculated as g extract/100 g dry material (d.m.).

The extraction pressure and the flow were maintained constant using a backpressure regulator. The extraction led to two different extraction yields, indicated as MLW-SCO_2_ and ML-SCO_2_, of 4 and 2% based on material collected from separators 1 and 2, respectively.

### 4.3. NMR Analysis of Extracts

One-dimensional and two-dimensional NMR spectra were obtained with a Bruker Avance III 400 Ultrashield spectrometer with a 400 MHz magnet. NMR spectra were acquired in deuterated chloroform (Sigma-Aldrich, Milan, Italy) with TMS as an internal standard. Duran^®^ 4.95 mm NMR tubes (Duran Group, Milan, Italy) were used. Chemical shifts are expressed in δ values in ppm. ^1^H-NMR, HSQC-DEPT, HMBC, COSY and ^13^C-NMR experiments were undertaken using standard Bruker sequences measuring p1 and d1 for each acquired sample. ML-SCO_2_ and MLW-SCO_2_ were dissolved (30 mg) in chloroform (1.5 mL) and used for NMR measurements.

### 4.4. Characterization of Volatile Constituents

The gas chromatographic analysis of the essential oils was performed with a Varian 3900 series gas chromatograph, fitted with a Saturn 2100T Ion Trap Mass spectrometer using a DB-5MS column (30 m × 0.25 mm × 0.25 μm). The instrument operated in electron impact mode at an ionization voltage of 70 eV. The injector temperature was set at 235 °C and the detector temperature at 230 °C, working in full scan mode. The oven temperature was initially programmed at 55 °C (isothermal for 5 min) and then increased to 250 °C at 4 °C/min and finally to 290 °C at 20 °C/min (isothermal for 5 min). The carrier gas was helium (99.9% purity) at a flow rate of 1 mL/min. Samples were dissolved in n-hexane and nonanol was added as internal standard (IS). To perform quantitative analysis, calibration curves were created mixing standard solutions of monoterpere (eucalyptol, thymol) and sesquiterpene (germacrene D) as reference compounds. Calibration curves were created plotting the amount of reference compound/amount of IS versus the area reference compound/area IS. The calibration curves were Y= 0.822x + 0.0324 for eucalyptol, Y= 0.782x + 0.0221 for thymol and Y= 0.7982x + 0.0108 for germacrene D. For quantification, a weighed amount of sample (50 mg exactly weighed) was diluted in 20 mL of n-hexane containing 100 ug/mL of nonanol. Solutions were injected with the split ratio 1/50. The identification of the essential oil constituents was performed by combining a comparison of mass spectral fragmentation patterns with those reported in NIST library, visual interpretation of the mass spectra and retention indices determined by reference to a homologous series of n-alkanes. Confirmation through injection of the reference standard was also performed when available. The identified constituents are listed in their order of elution in Table 1.

### 4.5. Gas Chromatography Mass Spectrometry (GC-MS) Analysis of Fatty Acid Methyl Esters

For GC-MS analysis of the fatty acids content, ML-SCO_2_ and MLW-SCO_2_ were derivatized with MeOH in the presence of H_2_SO_4_, leading to the esterification of fatty acids to fatty acid methyl esters, which offer excellent stability for GC analysis. Then, 150 mg of each extract was added to 15 mL of MeOH, 1 mL of CH2Cl2, 3 drops of H_2_SO_4_ and 25.7 mg of methylpentadecanoate (Sigma Aldrich, St. Louis, MO, USA), used as internal standard. The mixture was heated under reflux for 1 h and then cooled in an ice bath. A liquid/liquid partition was performed with 10 mL of water and 5 mL of diethyl ether, then the organic phase was collected and dried. The residue was re-dissolved with 1.5 mL of diethyl ether and put in a vial. GC-MS analysis was performed with an Agilent 7820A coupled to an Agilent 5977B MSD single quadrupole mass spectrometer, using an HP88 (60 m × 0.25 mm, 0.2 µm film thickness) as the stationary phase. Helium was the carrier gas with a column head pressure of 14.1 psi. The flow rate through the column was 1.19 mL/min. The injector was set at 300 °C with a split ratio of 20:1, the split flow was 23.9 mL/min and 1 µL injections were made. The temperature gradient started with an initial temperature of 120 °C before a linear increase to 240 °C at 3 °C/min. The total run time was 55 min. MS spectra were recorded in the range of m/z 40–650 using an EI ion source operating in positive ion mode.

### 4.6. Liquid Chromatography Atmospheric Pressure Chemical Ionization Mass Spectrometry (LC-APCI-MS) of Phytosterols

Quali-quantitative analysis of phytosterol derivatives was obtained by liquid chromatography atmospheric pressure chemical ionization mass spectrometry (LC-APCI-MS^n^) using a previously published method [17]. The measurements were performed with an Agilent 1260 chromatograph (Santa Clara, CA, USA) and a Varian MS-500 ion trap as detector. Separation was achieved using an Agilent Eclipse XDB C-8 (3.0 × 150 mm, 3.5 μm) as the stationary phase. The mobile phases were water (0.1%), formic acid (A) and acetonitrile (B). The elution gradient started at 90% A then decreased to 0% over 30 min; the flow rate was 0.5 mL/min. As reference compounds, beta sitosterol and stigmasterol were used, and standard solution was prepared at concentrations of 102.5 and 185 ug/mL in methanol. The sample injection volume was 10 µL. MS spectra were recorded in negative mode in the 50–2000 Da range, using an APCI ion source. Fragmentation of the main ionic species was obtained by the turbo data depending scanning (TDDS) function. Identification of compounds was obtained basing on fragmentation spectra as well as through the comparison of fragmentation patterns with data from the literature and injection of reference compounds. Quantification of phytosterols was obtained with the calibration curve method: beta sitosterol, y = 2.42 × 10^6^x − 1.16 × 10^7^, R² = 0.997; stigmasterol, y = 1.34 × 10^6^x + 1.42 × 10^7^, R² = 0.996.

### 4.7. Chromatographyc Isolation of Piperitone Oxide

A total of 300 mg of ML-SCO_2_ was diluted in 5 mL of ethyl acetate. Purification of piperitone oxide was performed by preparative TLC (silica gel 60 F254 on aluminum plate) with a mobile phase of n-exane/ethyl acetate of 1:2. Plates were developed, and spots were observed under an UV lamp. Relevant bands (F1–F6) were scrapped from the plate and compounds were eluted using methanol. Solutions were dried under nitrogen flow and fractions were analyzed with NMR. Band F-5 (20.5 mg) was identified as piperitone oxide on the basis of comparison of NMR spectra with data from the literature [25] while other fractions were mixtures of compounds.

### 4.8. In Vitro Experiments

#### 4.8.1. Reagents

Eagle’s minimum essential medium (MEM), trypsin-EDTA, penicillin, streptomycin, sodium pyruvate, L-glutamine, nonessential amino acid solution, fetal calf serum (FCS), plates and Petri dishes were purchased from EuroClone S.p.A. (Pero, Milan, Italy). Simvastatin was dissolved in physiological solution at 50 mM, filtered through a 0.22 µM filter and stored at −20 °C. Mint oil and mint wax were dissolved in 400 mg/mL of dimethyl sulfoxide (DMSO, Sigma-Aldrich Merck, Milan, Italy) as stock solution. Piperitone oxide was dissolved in DMSO to the final concentration of 80 mM. Simvastatin (Sigma-Aldrich Merck, Milan, Italy) was dissolved to a stock concentration of 50 mM in 0.1 M NaOH, and the pH was adjusted to 7.2 according to the manufacturer’s instructions. The solution was then sterilized by filtration.

#### 4.8.2. Cell Cultures

Human hepatic cancer cells Huh7 and HepG2 were cultured in MEM supplemented with 10% fetal bovine serum (FBS), 1% L-glutamine 200 mM, 1% sodium pyruvate 100X, 1% nonessential amino acids 100X and 1% penicillin/streptomycin solution (10.000 U/mL and 10 mg/mL, respectively) at 37 °C in a humidified atmosphere of 5% CO_2_ and 95% air. For the experiments, cells were incubated with indicated final concentrations in MEM/0.4%FBS. The final concentration of solvent (DMSO) did not exceed 0.5% *v/v* and the same amount was added to all of the experimental points in each assay.

#### 4.8.3. Cell Viability Assay

Cells were seeded in MEM/10% FBS in a 96-well tray at a cellular density of 8000 cells/well. The day after, cells were washed once with sterile phosphate buffered saline (PBS) and incubated with treatments (five experimental points for each compound; mint oil and wax: 62.5 µg/mL ÷ 1mg/mL, piperitone oxide: 12.5 µM ÷ 200 µM) for 72 h, after which the cell viability was evaluated by the sulforhodamine assay (SRB) according to a previously published protocol [26].

#### 4.8.4. Western Blot Analysis

Cells were seeded in MEM/10% FBS in a 6-well tray at a cellular density of 400,000 cells/well. The day after, cells were washed once with sterile PBS and incubated with the compounds at the indicated concentrations in DMEM/0.4% FBS. After 72 h of incubations, intracellular protein content was extracted in lysis buffer (50 mM Tris pH 7.5, 150 mM NaCl and 1% Nonidet-P40, containing 1% *v/v* of protease and phosphatase inhibitor cocktails). Protein samples (20 µg) and a molecular mass marker (Thermo Scientific, Waltham, MA, USA) were separated using 4–20% SDS-PAGE (Bio-Rad) under denaturation and reduction conditions. The protein samples were then transferred to a nitrocellulose membrane using the Trans-Blot^®^ Turbo™ Transfer System (Bio-Rad, Hercules, CA, USA) and nonspecific binding sites were blocked with a 5% nonfat dried milk Tris-buffered tween 20 (TBS-T20) solution, under agitation for 60 min at room temperature. The blots were incubated overnight at 4 °C with a diluted solution (5% nonfat dried milk) of anti-LDLR (rabbit polyclonal antibody, GeneTex GTX132860; dilution 1:1000), anti-PCSK9 (rabbit polyclonal antibody, GeneTex GTX129859; dilution 1:1000), anti-α-tubulin (mouse monoclonal antibody, clone DM1A, Sigma T6199; dilution 1:2000) and anti-GAPDH (rabbit polyclonal antibody, GeneTex GTX100118; dilution 1:3000). The membranes were washed with TBS-T20 and exposed for 90 min at room temperature to a diluted solution (5% nonfat dried milk) of the secondary antibodies (peroxidase-conjugate goat anti-rabbit and anti-mouse, Jackson ImmunoResearch, dilution 1:5000, cod. 111-036-045 and 115-036-062, respectively). Immunoreactive bands were detected by exposing the membranes to Clarity^TM^ Western Enhanced ChemiLuminescence (ECL) chemiluminescent substrates (Bio-Rad, Hercules, CA, USA) for 5 min, and images were acquired with an Azure c400 Imaging System (Aurogene, Rome, Italy). The densitometric readings were evaluated using ImageLab^TM^ software (Bio-Rad, Hercules, CA, USA).

#### 4.8.5. ELISA

Conditioned media were cleared by centrifugation (13,000 rpm for 10 min at 4 °C) and stored at −20 °C. The amount of PCSK9 was then quantified by using ELISA assays (R&D Systems, Minnesota, USA) according to the manufacturer’s instructions and as previously described [27].

#### 4.8.6. Statistical Analysis

Statistical analysis was performed using Prism statistical analysis package, version 5.01 (GraphPad Software, San Diego, CA, USA). When possible, *p*-values were determined by Student’s *t*-test. A probability value of *p* < 0.05 was considered statistically significant.

## 5. Conclusions

The extraction of *M. longifolia* leaves with supercritical fluid made it possible to obtain two different types of extracts, both of which were characterized for their chemical content in terms of fatty acids, phytosterols and terpenoids. MLW-SCO_2_ presented high amounts of unsaturated lipids (linoleic and alpha-linolenic acids at 2.8 and 9.4 mg/g, respectively) and phytosterols, with beta-sitosterol as the major component of these latter (5.6 mg/g). The extract also contained a significant amount of terpenoids. The ML-SCO_2_ fraction presented a limited amount of phytosterols but contained higher levels of terpenoids, with piperitone oxide being 26% on the basis of weight.

*In vitro* assays showed that ML-SCO_2_ induced a positive modulation of LDLR expression at a dose of 60 ug/mL, while MLW-SCO_2_ was inactive at the same concentration. The MLW-SCO_2_ extract at doses of 30 and 60 ug/mL completely inhibited PCSK9 expression. For this reason, the activity of isolated piperitone oxide was measured, and this compound was found to be a strong inhibitor of PCSK9 expression at doses of 12.5, 25 and 50 µM. The results suggested that the observed activity of the ML-SCO_2_ was not only related to piperitone oxide but that it was modulated by other phytoconstituents.

Overall, the outcome of the present study showed that supercritical fluid extraction of *M. longifolia* leaves results in a potential nutraceutical ingredient thanks to the presence of unsaturated fatty acids, phytosterols and terpenoids. Furthermore, significant activity was observed in the *in vitro* model used, demonstrating the ability of ML-SCO_2_ to modulate LDLR and PCSK9. Piperitone oxide was here reported for the first time as a significant inhibitor of PCSK9. Further investigations are needed on other monoterpenoids and related compounds to evaluate a possible new class of compounds with activity on PCSK9.

## Figures and Tables

**Table 1:** NMR key assignments for identification of main constituents of *M. longifolia* extracts.

Fatty Acid	δH	δC	HMBC	COSY
Terminal CH_3_	0.87 m	14.45	22.2 31.5 38.6	
CH_2_	1.21	29	29.6 22.0	
CH_2_ vicinal to carbonyl	2.29	28.5	178.5 28.9 24.4	2.78–2.00
sp^2^ CH	5.20	131.0 128.5	25.8	2.78-2.00
**Tymol**	δH	δC	HMBC	COSY
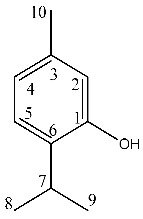		153 C-1		
	6.59 H-2	115 C-2	153(C-1) 131.5(C-6) 121.1(C-4) 21(C-10)	6.64 (H-4)
		135 C-3		
	6.64 H-4	121.1 C-4	131.5(C-6) 115(C-2) 21(C-10)	6.59 (H-2) 7.01 (H-5)
	7.01 H-5	126 C-5	153.5(C-1) 135(C-3) 26(C-7)	6.64 (H-4)
		131.5 C-6		
		26 C-7		
	1.19 CH_3_-8	22.4 C-8	131.5(C-6) 26(C-7)	
	1.19 CH_3_-9	22.4 C-9	131.5(C-6) 26(C-7)	
	2.22 CH_3_-10	21 C-10	135(C-3) 121.1(C-4) 115(C-2)	
**Piperitone Oxide**	δH	δC	HMBC	COSY
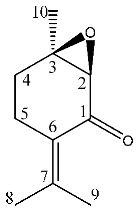		208.5 C-1		
	3.03 H-2	62.2 C-2	208.5(C-1) 61.5(C-3) 52(C-6) 21.5(C-10)	
		61.5 C-3		
	2.12 1.80 H-4	28 C-4	62.2(C-2) 52(C-6)	1.48 (H-5)
	1.48 1.52 H-5	17 C-5	208.5 (C-1) 61.5(C-3) 52(C-6)	
	1.83 H-6	52 C-6	208.5 (C-1) 29(C-7) 17(C-5)	
	2.33 H-7	29 C-7		
	0.90 CH_3_-8	20 C-8	52(C-6) 29(C-7) 18(C-9)	2.33(H-7)
	0.80 CH_3_-9	18 C-9	52(C-6) 29(C-7) 20(C-8)	2.33(H-7)
	1.40 CH_3_-10	21.1 C-10	62.2(C-2) 61.5(C-3) 28(C-4)	
**Piperitenone Oxide**	δH	δC	HMBC	COSY
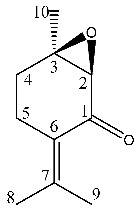		198 C-1		
	3.2 H-2	63.2 C-2	198(C-1) 129(C-6) 63.1(C-3) 21.7(C-10)	
		63.1 C-3		
		28 C-4		
	2.35 H-5	34 C-5	129(C-6) 63.1(C-3) 28(C-4)	
		129 C-6		
		149 C-7		
	2.07 CH_3_-8	23.3 C-8	149(C-7) 129(C-6) 23.3(C-9)	
	1.77 CH_3_-9	23.3 C-9	149(C-7) 129(C-6) 23.3(C-8)	
	1.40 CH_3_-10	21.7 C-10		
**Eucalyptol**	δH	δC	Correlation HMBC	COSY
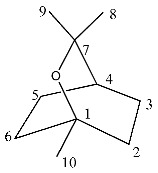		69.8 C-1		
		31.3 C-2		
		33.0 C-4		
		73 C-7		
	1.24 H-8	22.9 C-8	73(C-7) 33(C-4)	
	1.24 H-9	22.9 C-9	73(C-7) 33(C-4)	
	1.03 H-10	27.8 C-10	69.8(C-1) 31.3(C-2)	
**Germacrene D**	δH	δC	Correlation HMBC	COSY
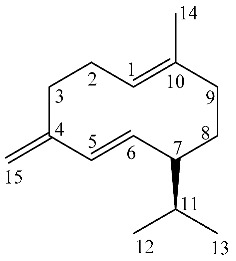		35 C-3		
		149 C-4		
	5.77 H-5	135 C-5	149(C-4) 52(C-7) 109(C15) 35(C-3)	
	5.21 H-6	133 C-6	149(C-4) 52(C-7) 26(C-8)	
		52 C-7		
		26 C-8		
	4.69/4.75 H-15	109 C-15	149(C-4) 135(C-5) 35(C-3)	

**Table 2:** Identification and quantification of volatile compounds in ML-SCO_2_ and MLW-SCO_2._

Nr	KI	Compounds	MW	ML-SCO_2_ % (*w/w*)	MLW-SCO_2_ % (*w/w*)
1	1031.9	eucalyptol	154.25	0.851 ± 0.094	1.059 ± 0.055
2	1130.4	trans-pinocarveol	158.28	0.029 ± 0.013	0.030 ± 0.020
3	1171.7	delta-terpineol	154.25	0.055 ± 0.033	0.021 ± 0.016
4	1194.1	alpha-terpineol	154.25	0.218 ± 0.112	0.064 ± 0.054
5	1208.7	p-Mentha-2,8-dien-1-ol	152.23	0.066 ± 0.050	0.043 ± 0.049
6	1232.8	carvenone	152.23	0.089 ± 0.034	0.037 ± 0.028
7	1259.0	trans-piperitone oxide	168.23	26.590 ± 3.465	16.497 ± 0.261
8	1273.8	gamma-diosphenol	168.23	0.067 ± 0.000	0.063 ± 0.046
9	1279.9	isopiperitenon	150.22	0.057 ± 0.040	0.002 ± 0.003
10	1289.8	5-ethyl 4-nonanone	170.29	0.785 ± 0.013	0.463 ± 0.068
11	1307.0	thymol	150.22	1.23 ± 0.009	0.472 ± 0.098
12	1321.8	5-ethyl-4-tridecanone	226.4	0.548 ± 0.021	0.298 ± 0.044
13	1370.9	piperitenone oxide	166.22	3.775 ± 0.208	1.869 ± 0.102
14	1385.8	beta-bourbonene	204.35	0.132 ± 0.013	0.068 ± 0.059
15	1392.7	alpha-guaiene	204.35	0.048 ± 0.006	0.070 ± 0.012
16	1420.7	beta-caryophyllene	204.35	0.329 ± 0.067	0.292 ± 0.051
17	1450.3	p-menthane-1,3-diol	172.26	0.044 ± 0.040	0.005 ± 0.004
18	1457.2	alpha-caryophyllene	204.35	0.009 ± 0.009	0.012 ± 0.009
19	1462.2	1-acetoxy-p-menth-3-one	212.28	0.289 ± 0.037	0.155 ± 0.095
20	1485.9	germacrene D	204.35	3.085 ± 0.420	2.600 ± 0.317
21	1501.3	gamma-elemene	204.35	0.184 ± 0.045	0.103 ± 0.069
22	1509.5	ND		0.093 ± 0.083	0.032 ± 0.026
23	1528.6	gamma-cadinene	204.35	0.044 ± 0.025	0.065 ± 0.021
24	1579.3	spathulenol	220.35	0.363 ± 0.001	0.550 ± 0.156

**Table 3:** Fatty acid composition of *M. longifolia* extracts.

Fatty Acids	ML-SCO_2_ % (*w/w*)	MLW-SCO_2_ % (*w/w*)
Palmitic acid	2.84 ± 0.08	3.68 ± 0.08
Stearic acid	0.38 ± 0.02	0.50 ± 0.01
Oleic acid	0.50 ± 0.03	0.61 ± 0.03
Linoleic acid	2.18 ± 0.04	2.81 ± 0.09
Arachic acid	0.40 ± 0.01	0.59 ± 0.04
Alfa-linolenic acid	1.39 ± 0.01	9.39 ± 0.05
Docosanoic acid	nd	0.27 ± 0.01

**Table 4:** Phytosterol composition of *M. longifolia* extracts.

tr	*m/z*	Compounds	ML-SCO_2_ (mg/g)	MLW-SCO_2_ (mg/g)
32.3	397.5	β-sitosterol	0.31 ± 0.03	5.57 ± 0.03
31.7	397.5	Gamma-sitosterol	0.03 ± 0.01	0.32 ± 0.01
29.9	395.5	Stigmasterol	0.05 ± 0.01	0.99 ± 0.01
28.2	395.5	Fucosterol	0.03 ± 0.01	0.50 ± 0.01
25.8	381.5	Brassicasterol	-	0.06 ± 0.01
23.6	409	Cycloartanol	-	0.07 ± 0.01
		TOT	0.41 ± 0.02	7.50 ± 0.03
